# WILLINGNESS TO LIVE UNDER IMPAIRMENT: FACTORS ASSOCIATED WITH DESIRED LONGEVITY

**DOI:** 10.1093/geroni/igae098.0371

**Published:** 2024-12-31

**Authors:** Clara Paula de Couto, Fiona Rupprecht, Jana Nikitin, Klaus Rothermund

**Affiliations:** Friedrich Schiller University Jena, Jena, Thuringen, Germany; University of Vienna, Vienna, Wien, Austria; University of Vienna, Vienna, Wien, Austria; Friedrich Schiller University Jena, Jena, Thuringen, Germany

## Abstract

This study delves into the complexities of individuals’ desires for longevity and their willingness to live with impairment. When deciding about their longevity desires, some individuals may focus on present-oriented, concrete aspects of their lives, like their current state of health, whereas others may weigh up more future-oriented, abstract aspects, such as how important it is to be healthy. We investigated what factors are associated with individuals’ willingness to live with impairment. Factors associated with willingness to live with impairment were examined in a sample of N = 790 German participants aged 39–90 years (Mage = 63.38, SD = 14.30, 51% female). To examine age-specific factors related to desired longevity under impairment conditions, chronological age was added as a moderator in the analyses. Findings indicated that for middle-aged adults, fear of becoming a burden and higher importance of health were associated with lower willingness to live with impairment. For older adults, lower willingness to live with impairment was associated with higher levels of instrumental preparation for the end of life. The obtained age differences can be understood as reflecting a shift in time perspective and personal experiences. Among middle-aged adults, willingness to live with impairment is more strongly influenced by distant events that pertain to the future (e.g., fear of becoming a burden). Older adults, however, place more importance on their current life situation (e.g., instrumental preparation), and their willingness to live with impairment seems to be more conditional on practical aspects in the present.

